# Intraluminal instillation of urokinase and autologous plasma: a method to unblock occluded central venous ports

**DOI:** 10.1186/1471-2407-6-103

**Published:** 2006-04-24

**Authors:** Georg Seifert, Hanno Riess, Karl Seeger, Guenter Henze, Anja Borgmann

**Affiliations:** 1 Department of Paediatric Oncology/Haematology, Charité – Universitätsmedizin Berlin, Germany; 2 Department of Haematology and Oncology, Charité – Universitätsmedizin Berlin, Germany

## Abstract

**Background:**

Therapeutic use and effective function of recombinant urokinase (r-UK) for occluded ports need the presence of plasminogen.

**Methods:**

As a therapeutic proof of principle, we demonstrate that the use of r-UK and autologous plasma effectively reestablishes the function of occluded central venous ports (CVP) resistant to routine management of catheter occlusion. Five patients with occluded ports resistant to the routine management were treated.

**Results:**

All patients were successfully treated with thrombolytic therapy using intraluminal instillation of r-UK and autologous plasma.

**Conclusion:**

Instillation of r-UK and autologous plasma is a safe and effective method for management of CVP occlusion.

## Background

**Figure 1:**
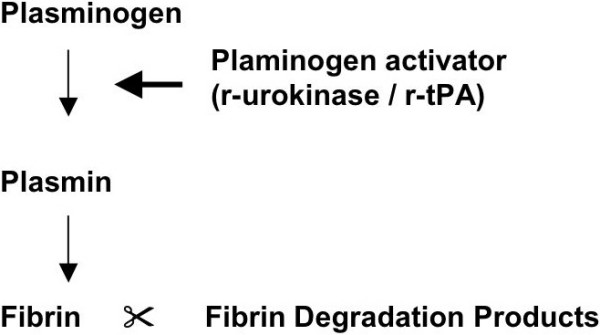
Fibrinolytic System.

Regular application of plasma products or chemotherapy is often complicated by catheter-related problems such as blockage. Especially in children, an effective and simple reestablishment of catheter patency is important. Maintenance of catheter function is usually achieved with flushing (sodium chloride and/or heparin) or urokinase instillation [1,2]. In the majority of cases low-dose urokinase therapy is a safe and efficacious treatment for catheter occlusion, frequently obviating the need for catheter removal. However, in some cases of occluded central venous ports (CVP), r-UK instillation is not effective. Because effective therapeutic function of r-UK requires the presence of plasminogen (see Figure 1) contained in human plasma, we combined autologous plasma and r-UK for treatment of occluded CVP in adult and paediatric patients.

## Methods

Five patients with occluded CVP were treated. Patient characteristics are given in Table 1. After up to eight attempts every 15 minutes with drawing and pressing to remove the CVP occlusion with sodium chloride and/or heparin and subsequently with 10 mg recombinant tissue plasminogen activator (r-tPA) and 5.000 IU r-UK, we used a combination of 2 ml of the patient's plasma and 10.000 IU r-UK (Medac GmbH, Germany) dissolved in 1 ml sodium chloride. The plasma was obtained by centrifugation at 1100 g for 10 minutes in a sterile syringe. Next, the combination of autologous plasma and r-UK were slowly mixed for 2 minutes within a 5 ml- syringe before application. The combination was slowly instilled, whereby a volume slightly above the CVP volume was injected. Thirty minutes after instillation, success was assessed by attempting to aspirate the combination and residual clot with an empty, sterile syringe. Up to three further attempts of aspiration were made. If the aspiration was successful, the catheter lumen was immediately flushed with 10 ml sodium chloride and/or heparin. Informed consent was obtained from patients, and, if necessary, from parents, as approved by the Institutional Ethical Committee.

## Results

We report on five patients with an CVP occlusion that could not be managed by routine procedures. All patients had internal jugular CVPs, which had all been implanted in a surgical unit. The indications for CVP placement were regular infusion of plasma products, chemotherapy and antibiotics. We tested the new method described in this paper as a last resort before catheter replacement. All instillations resulted in successful reestablishment of catheter function after approximately 60 minutes of urokinase/plasma instillation. No patient experienced adverse effects during or after instillation. No bleeding complications occurred. The entire management procedure was easy to perform and well tolerated. In one case the instillation of urokinase and plasma failed. The explanted CVP showed that a silicon particle, which had originated during puncture, had occluded the CVP.

## Discussion

Catheter occlusion is a common complication of long-term CVP placement. Therapeutic fibrinolytic agents like r-UK or r-tPA are used successfully to restore function of occluded CVP [1,2] Generally one or two instillationsof r-UK are required in the majority of patients. A 98.1% patency rate is reported when treating catheter occlusions in paediatric patients with r-UK at a concentration of 5,000 IU/mL [4] However, after repeated applications of plasminogen activators, a clot may become deprived of plasminogen. This will result in the failure of further attempts.

## Conclusion

The method we have described in this paper is useful in such situations. We were able to avoid catheter replacement in all five patients with thrombotic catheter occlusions that were treated with r-UK and autologous plasma. This is especially important for children.

## Competing interests

The author(s) declare that they have no competing interests.

## Authors' contributions

Georg Seifert was involved in acquisition of data, drafted the manuscript and has given final approval of the version to be published.

Hanno Riess was involved in acquisition of data and made substantial contributions to conception and design and has given final approval of the version to be published.

Karl Seeger was involved in acquisition of data and critically revised the manuscript and has given final approval of the version to be published.

Günter Henze critically revised the manuscript and has given final approval of the version to be published.

Anja Borgmann was involved in acquisition of data and has given final approval of the version to be published.

## Pre-publication history

The pre-publication history for this paper can be accessed here:



## Figures and Tables

**Table 1:** Patient characteristics.

**Initials**	**Age (years)**	**Diagnosis**	**Therapy**
NK	6	Hemophilia	Factor VIII
AH	3	Hemophilia	Factor VIII
HR	67	Hepatocellular Carcinoma	Fluorouracil, Folic Acid
MA	51	Colon Carcinoma	Fluorouracil, Folic Acid
BH	53	Mammary Carcinoma	Fluorouracil, Doxorubicin
